# Supplementary material for: Molecular basis for the production of cyclic peptides by plant asparaginyl endopeptidases
Source: Nat Commun. 2018 Jun 20;9:2411. doi: 10.1038/s41467-018-04669-9 (PMC6010433; doi:10.1038/s41467-018-04669-9)
Supplement: Supplementary file 1 — Supplementary Information [file 41467_2018_4669_MOESM1_ESM.pdf]

Supplementary Information

Jackson et al. **Molecular basis for the production of cyclic peptides by plant asparaginyl endopeptidases**

a

Character	Violaceae	Fabaceae	Cucurbitaceae	Rubiaceae	Solanaceae
Gene origin	Specific	Albumin	Specific	Specific	Specific
N-term domains	Multi/Singletons	None	Multi	Multi/Singletons	Singleton
Cyclotide domains	Multi/Singletons	Singleton	Multi	Multi/Singletons	Singleton
Topologies present	Bracelet/Möbius/Hybrid	Bracelet/Möbius/Hybrid	Squash-TI	Bracelet/Möbius/Hybrid	Bracelet

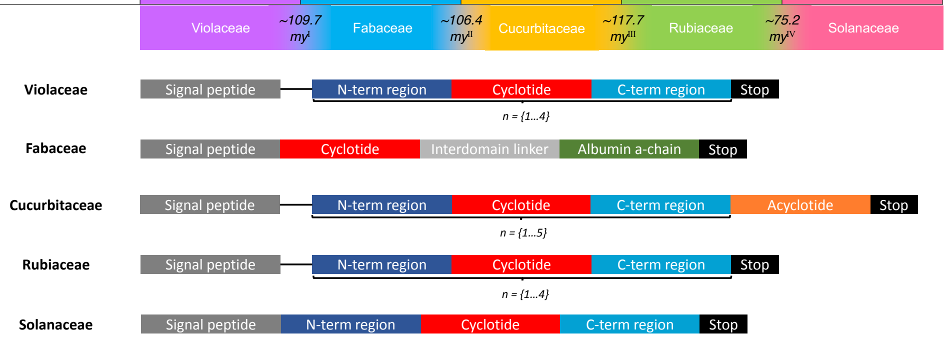

I. Divergence of Malpighiales; II. Most recent common ancestor between Rosales – (Cucurbitales + Fagales);
III. Divergence of Saxifragales; IV. Most recent common ancestor between Gentianales - Solanales

b

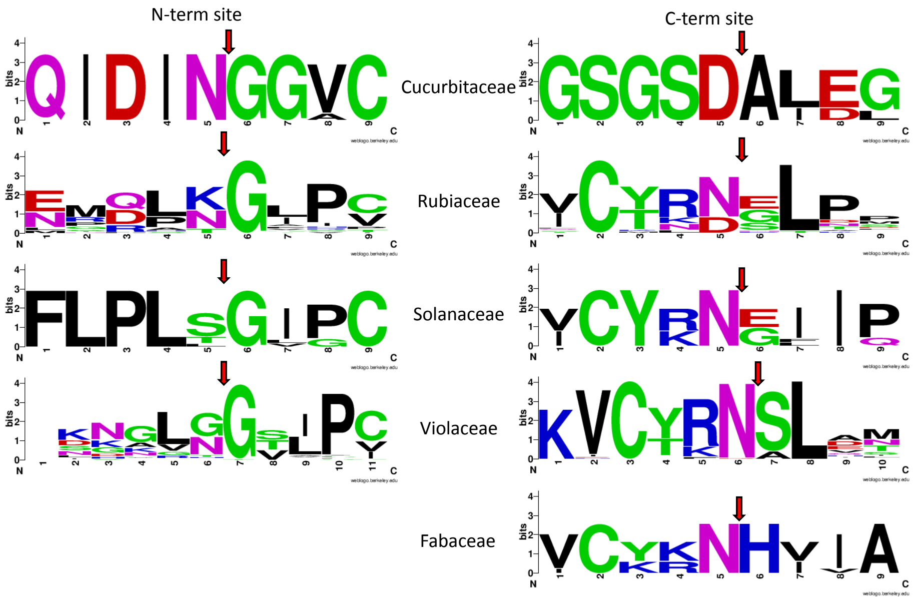

Supplementary Figure 1. Summary of cyclotide-encoding gene diversity. a, Attributes of cyclotide precursors and domains across five plant families. All cyclotide encoding genes discovered in the Violaceae, Cucurbitaceae, Rubiaceae, and Solanaceae exist as dedicated expression units, whereas in the Fabaceae, cyclotides are encoded within an albumin gene[1,2]. All precursors possess a signal peptide that directs biosynthesis of cyclotides into the plant cells endomembrane system. Cleavage at the amino terminus of the cyclotide domain must occur first in order to free up the N-terminus for an AEP mediated transpeptidation reaction with the C-terminus of the nascent cyclotide. With the exception of cyclotides in the Solanaceae and Fabaceae, cyclotides domains are often encoded as repeated units. Mature cyclotides are classified into either the Möbius, bracelet or trypsin inhibitor like sub families, where the Möbius and bracelets are characterised by the presence or absence of a twist in the backbone respectively[3], with the trypsin inhibitor class categorised by function. Estimates of evolutionary time between cyclotide producing clades as provided by [4]. For Malpighiales (contains Violaceae) and Saxifragales (contains Fabaceae), divergence time estimates are for that order. Most recent common ancestor estimates are for the last common ancestor of II Fabaceae and Curcubitaceae, and IV Rubiaceae and Solanaceae. **b,** N- and C-terminal precursor sequence diversity across cyclotide-producing plant families. N-terminal processing sites are diverse, with flanking residues suggesting cleavage by AEPs or enzymes with trypsin-like activity. In the Fabaceae, the N-terminus is cleaved by a signal peptidase during translation into the ER thus no N-terminal logo is given. At the C-terminus, an Asx residue is required for transpeptidation by AEP. Other residues appear conserved before the Asx residue (ex: Tyr, Arg, Lys), and after the Asx (ex. Gly).

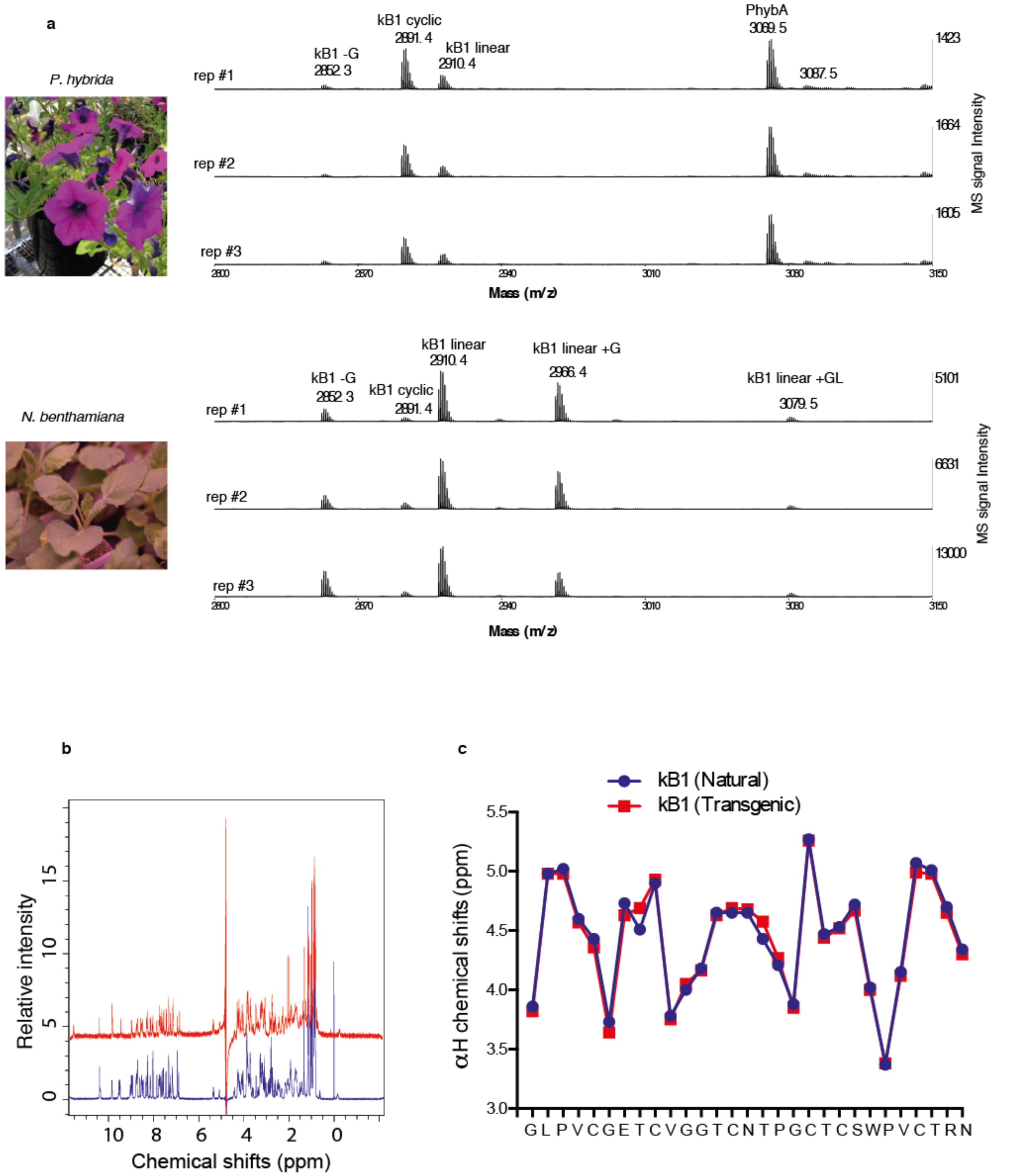

Supplementary Figure 2. Petunia correctly processes *Oak1* into predominant cyclic kB1. a, MALDI-MS analysis of petunia and *N. benthamiana* produced peptides upon expression of *Oak1*. Predominant peptide mass signals for cyclic kB1 (2891.4 m/z) are evident in petunia leaf extracts whilst predominant linear kB1 related peptides (2910.4, 2966.4, 3079.5 m/z) are evident in *N. benthamiana* leaf extracts. The peptide mass of 3069.5 m/z in petunia leaf extracts represents the endogenous cyclotide PhybA[5]. **b**, Comparative NMR analysis of *oak1* transgene derived kB1 extracted from petunia with kB1 extracted from *O. affinis* plants. Purified kB1 from petunia leaf (in red) has an identical (b) 1D NMR spectra and (c) αH chemical shifts to that of native kB1 from *O. affinis* (in blue).

```
ResAlign   1        10        20        30        40        50        60        70        80
PxAEP1     MIRYVATTLFLIGLSLNIFVSESRNVLRLPSEVSRFFGADESVR-NKDDDSV-GTRWAILLAGSNGYWNYRHQADICHAY
PxAEP2     MIGS-A--LLIIGLSI-LAAVDGRDVLKLPSEASKFF-SE---KYG--DGSVEGTRWGVLLAGSRGYWNYRHQADVCHAY
PxAEP3a    MI-NVAGILILVGFSI-IAAGEGRNVLKLPSEASRFF--D---K-G-DDDSV-GTRWAVLLAGSNGYWNYRHQADVCHAY
PxAEP3b    MISHVAGILILVGFSI-LGAGEGRNVLKLPSEASRFF--K---K-GEDDDSV-GTRWAVLLAGSNSYWNYRHQADVCHAY
poly #     **   *  *    * *    * ** **** * **        * ** ****  *****  ********* ****

ResAlign            90       100       110       120       130       140       150       160
PxAEP1     QLLKKGGLKDENIVVFMYDDIANNEENPRPGIIINSPHGEDVYKGVPKDYTGDDVTVDNFLAVILGNKAALSGGSGKVVN
PxAEP2     QLLKKGGLKDENIIVFMYDDIANNYENPRPGIIINSPDGEDVYKGVPKDYTGHNVTVNNFLAVILGDKAALTGGSGKVVE
PxAEP3a    QLLRKGGLKDENIIVFMYDDIAYNEENPRKGVIINSPAGEDVYKGVPKDYTGDDVNVDNFLAVLLGNKTALTGGSGKVVD
PxAEP3b    QLLRKGGLKDENIVVLMYDDIAYNEENPRKGVIINNPAGEDVYKGVPKDYTGDDVNVDNFLAVLLGNKTAITGGSGKVVD
poly #     *** *********  ****** * **** * ***  **************   * * ***** ** * *  *******

ResAlign           170       180       190       200       210       220       230       240
PxAEP1     SGPNDHIFIYYSDHGGPGVLGMPTDPYLYANDLIDVLKKKHASGTYKSLVFYLEACESGSIFEGLLPEGLNIYATTASNA
PxAEP2     SGPNDHIFIFYSDHGGPGVLGMPTYPNLYADELIDALKRKHASGTYKSLVFYIEACESGSIFEGLLPEGLNIYATTASNA
PxAEP3a    SGPNDHIFVFYSDHGGPGVLGMPTNPYLYASDLIGALKKKHASGTYKSLVLYIEACESGSIFEGLLPEGLNVYATTASNA
PxAEP3b    SGPNDHIFIFYTDHGGPGVLGMPTKPYLYASDLIGALKKKHASGTYKSLVLYVEACEAGSIFEGLLPEGLNVYATTASDA
poly #     ********  ************** *** ** ** ***********  ***** ************* ******* *

ResAlign           250       260       270       280       290       300       310       320
PxAEP1     EESSWGTYCPGEYPSPPIEYETCLSDLYSIAWMEDSDIHNLRTESLKQQYHLVKDRTANGNPFYGS-HVMQYGDLNLSKN
PxAEP2     EEDSWATYCPGDNQSPPPEYQTCLGDLYSVSWMEDSEKHDLQTETLGMQYELVRRRTANSFP-FASSHVMQYGDLKLRDD
PxAEP3a    VESSWGTYCPGENPSPPPEYETCLGDLYAVSWMEDSEKHNLQTESLRQQYHLVKRRTANGNSAYGS-HVMQFGDLKLSVD
PxAEP3b    VEGSWVTYCPGQNPSPPPEYTTCLGDLYSVSWMEDSEKHNLQTESLRQQYHLVKEKIA-----YAS-HVMQYGDLKLSMD
poly #       *  ***  *****    ***** *** ***    *****  *  * ** *  **        *  ** ** *** *

ResAlign           330       340       350       360       370       380       390       400
PxAEP1     PLFVYMGTNPANDNYTFGADNSLRVS-KVVNQRDADLLHFWYKFRKAPEGSARKFEAQKQLNEAISHRMHLDNSIALVGK
PxAEP2     PISLYMGTNPANYTYSFLDENSL-LSSKPVNQRDADLLHFWEKFLKARQGSARKLEAQKQLTEAMTHRMHIDDSITLVGK
PxAEP3a    SLSMYMGTDPANDNSTFVDDNSLGASSKAVNQRDADLLHFWDKFLKAPEGSARKVEAQKQFTEAMSHRMHLDNSMALVGK
PxAEP3b    SLSMYMGTDPANDNYTFVDDNSLGTSSKAVNQRDADLLHFSDKFLKAPEGSARKVEAQKQFAEAMSHRLHLDNSMALVGK
poly #         **** ***  *   *** ** * *********** ** **  ***** *****  **   *** * *  ****

ResAlign           410       420       430       440       450       460       470       480
PxAEP1     LLFGIKNVPEVLSSVRPAGQPLVDDWDCLKSYVRTFETHCGSLSQYGMKHMRSIANICNAGIKMEQMVEASAQACPRVPS
PxAEP2     LLFGIEKGTEELTRVRPSGEPLVDDWDCLKSFVGTFETYCGSLSQYGLKYMRAIANICNASIKVEQMAKASAQACVDVPS
PxAEP3a    LLFGIQKGPEVLKRVRSDGQPLVDDWACLKSFVRTFETHCGSLSQYGMKHMRSIANICNAGIKMEQMVEASSQACPSVPS
PxAEP3b    LLFGIKKGPEVLKRVRSDGQLLVDDWACLKSFVRTFETHCGSLSQYGMKHMRSFANICNAGIKVEQMVEASSQACPSVPS
poly #     *****    * *  **  *  ****** **** * **** ******** * **  ****** ** ***   ** ***  ***

ResAlign           490
PxAEP1     NTWSSLHRGFSA
PxAEP2     NSWDSLDEGFSA
PxAEP3a    NTWSSLHRGFSA
PxAEP3b    NTWSSLHRGFSA
poly #     * * **  ****
```

Supplementary Figure 3. Alignment of petunia AEP isoforms. Protein sequences were aligned using ClustalW[6]. Identical residues are marked with a star and similar residues with a dot. Putative signal peptides predicted by SignalP 4.0[7] are italicized with putative propeptide cleavage sites indicated by arrows and proposed based on[8]. Boxed residues indicate putative N-terminal vacuole targeting signals as predicted by[9]. The catalytic histidine and cysteine residues are highlighted in yellow. The residue homologous to the Gatekeeper residue of OaAEP1$_b$[8] is shown in green with the cysteine flanking poly-proline loop in blue. The marker of ligase activity (MLA) (this study) is shown in magenta. All polymorphic residues between PxAEP3a and PxAEP3b are underlined.

```
OaAEP1b                     1 MVRYLAGAVLLLVVLSVAAAVSGARDGDYLHLPSEVSRFFRPQETNDDHGEDSVGTRWAVLIAGSKGYANYRHQAGVCHA  80
OaAEP1b_MLA                 1 MVRYLAGAVLLLVVLSVAAAVSGARDGDYLHLPSEVSRFFRPQETNDDHGEDSVGTRWAVLIAGSKGYANYRHQAGVCHA  80
OaAEP2                      1 MVRYPAGAVLLLVVLSVVA-VDGARDG-YLKLPSEVSDFFRPRNTND--GDDSVGTRWAVLLAGSNGYWNYRHQADLCHA  76
OaAEP2+                     1 MVRYPAGAVLLLVVLSVVA-VDGARDG-YLKLPSEVSDFFRPRNTND--GDDSVGTRWAVLLAGSNGYWNYRHQADLCHA  76
OaAEP2_select               1 MVRYPAGAVLLLVVLSVVA-VDGARDG-YLKLPSEVSDFFRPRNTND--GDDSVGTRWAVLLAGSNGYWNYRHQADLCHA  76
OaAEP2_pocket_poly_MLA      1 MVRYPAGAVLLLVVLSVVA-VDGARDG-YLKLPSEVSDFFRPRNTND--GDDSVGTRWAVLLAGSNGYWNYRHQADLCHA  76
OaAEP2_pocket_MLA           1 MVRYPAGAVLLLVVLSVVA-VDGARDG-YLKLPSEVSDFFRPRNTND--GDDSVGTRWAVLLAGSNGYWNYRHQADLCHA  76
OaAEP2_MLA                  1 MVRYPAGAVLLLVVLSVVA-VDGARDG-YLKLPSEVSDFFRPRNTND--GDDSVGTRWAVLLAGSNGYWNYRHQADLCHA  76
OaAEP2_pocket               1 MVRYPAGAVLLLVVLSVVA-VDGARDG-YLKLPSEVSDFFRPRNTND--GDDSVGTRWAVLLAGSNGYWNYRHQADLCHA  76
                              **** ************ * * ***** **.****** ****. ***  *.**********.*** ** ****** .***

OaAEP1b                    81 YQILKRGGLKDENIVVFMYDDIAYNESNPRPGVIINSPHGSDVYAGVPKDYTGEEVNAKNFLAAILGNKSAITGGSGKVV 160
OaAEP1b_MLA                81 YQILKRGGLKDENIVVFMYDDIAYNESNPRPGVIINSPHGSDVYAGVPKDYTGEEVNAKNFLAAILGNKSAITGGSGKVV 160
OaAEP2                     77 YQILKRGGLKDENIVVFMYDDIAYNEENPRPGVIINSPHGSDVYAGVPKDYTGDQVNAKNFLAAILGNKSAITGGSGKVV 156
OaAEP2+                    77 YQILKRGGLKDENIVVFMYDDIAYNEENPRPGVIINSPHGSDVYAGVPKDYTGDQVNAKNFLAAILGNKSAITGGSGKVV 156
OaAEP2_select              77 YQILKRGGLKDENIVVFMYDDIAYNEENPRPGVIINSPHGSDVYAGVPKDYTGDQVNAKNFLAAILGNKSAITGGSGKVV 156
OaAEP2_pocket_poly_MLA     77 YQILKRGGLKDENIVVFMYDDIAYNEENPRPGVIINSPHGSDVYAGVPKDYTGDQVNAKNFLAAILGNKSAITGGSGKVV 156
OaAEP2_pocket_MLA          77 YQILKRGGLKDENIVVFMYDDIAYNEENPRPGVIINSPHGSDVYAGVPKDYTGDQVNAKNFLAAILGNKSAITGGSGKVV 156
OaAEP2_MLA                 77 YQILKRGGLKDENIVVFMYDDIAYNEENPRPGVIINSPHGSDVYAGVPKDYTGDQVNAKNFLAAILGNKSAITGGSGKVV 156
OaAEP2_pocket              77 YQILKRGGLKDENIVVFMYDDIAYNEENPRPGVIINSPHGSDVYAGVPKDYTGDQVNAKNFLAAILGNKSAITGGSGKVV 156
                              ************************** **************************..*************************

OaAEP1b                   161 DSGPNDHIFIYYTDHGAAGVIGMPSKPYLYADELNDALKKKHASGTYKSLVFYLEACESGSMFEGILPEDLNIYALTSTN 240
OaAEP1b_MLA               161 DSGPNDHIFIYYTDHGAAGVIGMPSKPYLYADELNDALKKKHASGTYKSLVFYLEACESGSMFEGILPEDLNIYALTSTN 240
OaAEP2                    157 NSGPNDHIFIYYTDHGGPGVLGMPVGPYIYADDLIDTLKKKHASGTYKSLVFYLEACESGSMFEGLLPEGLNIYATTASN 236
OaAEP2+                   157 DSGPNDHIFIYYTDHGGPGVLGMPVKPYIYADDLIDTLKKKHASGTYKSLVFYLEACESGSMFEGLLPEGLNIYATTASN 236
OaAEP2_select             157 DSGPNDHIFIYYTDHGGPGVLGMPVKPYIYADDLIDTLKKKHASGTYKSLVFYLEACESGSMFEGLLPEGLNIYATTASN 236
OaAEP2_pocket_poly_MLA    157 NSGPNDHIFIYYTDHGGPGVLGMPVGPYIYADDLIDTLKKKHASGTYKSLVFYLEACESGSMFEGLLPEGLNIYATTASN 236
OaAEP2_pocket_MLA         157 NSGPNDHIFIYYTDHGGPGVLGMPVGPYIYADDLIDTLKKKHASGTYKSLVFYLEACESGSMFEGLLPEGLNIYATTASN 236
OaAEP2_MLA                157 NSGPNDHIFIYYTDHGGPGVLGMPVGPYIYADDLIDTLKKKHASGTYKSLVFYLEACESGSMFEGLLPEGLNIYATTASN 236
OaAEP2_pocket             157 NSGPNDHIFIYYTDHGGPGVLGMPVGPYIYADDLIDTLKKKHASGTYKSLVFYLEACESGSMFEGLLPEGLNIYATTASN 236
                              **************   **.***   **.***.* *.****************************.*** ***** *..*

OaAEP1b                   241 TTESSWCYYCPAQENP-PPPEYNVCLGDLFSVAWLEDSDVQNSWYETLNQQYHHVDKRIS-----HASHATQYGNLKLGE 314
OaAEP1b_MLA               241 TTESSWCYYCPAQENP-PPPEYNVCLGDLFSVAWLEDSDVQNSWYETLNQQYHLVKARTSNGNSAYASHATQYGNLKLGE 314
OaAEP2                    237 AEESSWGTYCPGEY-PSPPPEYDTCLGDLYSVAWMEDSEVHNLRSETLKQQYHLVKARTSNGNSAYGSHVMQYGDLKLSV 315
OaAEP2+                   237 AEESSWCYYCPGQY-ASPPPEYDVCLGDLYSVAWMEDSEVHNLRSETLKQQYHHVKARTS-----YGSHVMQYGDLKLSE 310
OaAEP2_select             237 AEESSWCYYCPGQEYPSPPPEYDVCLGDLYSVAWMEDSEVHNLRSETLKQQYHHVDKRIS-----HASHVMQYGDLKLSE 311
OaAEP2_pocket_poly_MLA    237 AEESSWCYYCPGQEYPSPPPEYDVCLGDLYSVAWMEDSEVHNLRSETLKQQYHHVDKRIS-----HASHVMQYGDLKLSV 311
OaAEP2_pocket_MLA         237 AEESSWCYYCPGEY-PSPPPEYDTCLGDLYSVAWMEDSEVHNLRSETLKQQYHHVDKRIS-----HASHVMQYGDLKLSV 311
OaAEP2_MLA                237 AEESSWGTYCPGEY-PSPPPEYDTCLGDLYSVAWMEDSEVHNLRSETLKQQYHHVDKRIS-----HASHVMQYGDLKLSV 311
OaAEP2_pocket             237 AEESSWCYYCPGEY-PSPPPEYDTCLGDLYSVAWMEDSEVHNLRSETLKQQYHLVDARTS-----YGSHVMQYGDLKLSV 311
                              . ****  ***   .    *****  *****.****.***.*.*    *** **** *  * *        **  *** ***

OaAEP1b                   315 EGLFVYMGSNPANDNYTSLDGNALTPSSIVVNQRDADLLHLWEKFRKAPEGSARKEEAQTQIFKAMSHRVHIDSSIKLIG 394
OaAEP2                    316 DNLFLYMGTNPANDNYTFVDDNALRPSSKAVNQRDADLLHFWDKFRKAPEGSARKEEARKQVFEAMSHRMHIDNSIKLVG 395
OaAEP2+                   311 DGLFLYMGTNPANDNYTFVDDNALRPSSKAVNQRDADLLHFWDKFRKAPEGSARKEEARKQVFEAMSHRMHIDNSIKLVG 390
OaAEP2_select             312 DGLFLYMGSNPANDNYTFVDDNALRPSSKAVNQRDADLLHFWDKFRKAPEGSARKEEARKQVFEAMSHRMHIDNSIKLVG 391
OaAEP2_pocket_poly_MLA    312 DNLFLYMGSNPANDNYTFVDDNALRPSSKAVNQRDADLLHFWDKFRKAPEGSARKEEARKQVFEAMSHRMHIDNSIKLVG 391
OaAEP2_pocket_MLA         312 DNLFLYMGSNPANDNYTFVDDNALRPSSKAVNQRDADLLHFWDKFRKAPEGSARKEEARKQVFEAMSHRMHIDNSIKLVG 391
OaAEP2_MLA                312 DNLFLYMGSNPANDNYTFVDDNALRPSSKAVNQRDADLLHFWDKFRKAPEGSARKEEARKQVFEAMSHRMHIDNSIKLVG 391
OaAEP2_pocket             312 DNLFLYMGSNPANDNYTFVDDNALRPSSKAVNQRDADLLHFWDKFRKAPEGSARKEEARKQVFEAMSHRMHIDNSIKLVG 391
                              ..**.***.********  .* *** ***   ********** *.***************. *.* *****.*** ****.*

OaAEP1b                   395 KLLFGIEKCTEILNAVRPAGQPLVDDWACLRSLVGTFETHCGSLSEYGMRHTRTIANICNAGISEEQMAEAASQACASIP 474
OaAEP2                    396 KLLFGIERGAEILDAVRPAGQPLADDWTCLKSLVRTFETHCGSLSQYGMKHMRTIANICNAGITKEQMAEASAQACSSVP 475
OaAEP2+                   391 KLLFGIERGAEILDAVRPAGQPLADDWTCLKSLVRTFETHCGSLSQYGMKHMRTIANICNAGITKEQMAEASAQACSSVP 470
OaAEP2_select             392 KLLFGIERGAEILDAVRPAGQPLADDWTCLKSLVRTFETHCGSLSQYGMKHMRTIANICNAGITKEQMAEASAQACSSVP 471
OaAEP2_pocket_poly_MLA    392 KLLFGIERGAEILDAVRPAGQPLADDWTCLKSLVRTFETHCGSLSQYGMKHMRTIANICNAGITKEQMAEASAQACSSVP 471
OaAEP2_pocket_MLA         392 KLLFGIERGAEILDAVRPAGQPLADDWTCLKSLVRTFETHCGSLSQYGMKHMRTIANICNAGITKEQMAEASAQACSSVP 471
OaAEP2_MLA                392 KLLFGIERGAEILDAVRPAGQPLADDWTCLKSLVRTFETHCGSLSQYGMKHMRTIANICNAGITKEQMAEASAQACSSVP 471
OaAEP2_pocket             392 KLLFGIERGAEILDAVRPAGQPLADDWTCLKSLVRTFETHCGSLSQYGMKHMRTIANICNAGITKEQMAEASAQACSSVP 471
                              *******. .*** ********* ***.**.*** **********.***.* ***********. ******..***.*.*

OaAEP1b                   475 *              475
OaAEP2                    476 SNPWSSLHKGFSA* 489
OaAEP2+                   471 SNPWSSLHKGFSA* 484
OaAEP2_select             472 SNPWSSLHKGFSA  484
OaAEP2_pocket_poly_MLA    472 SNPWSSLHKGFSA  484
OaAEP2_pocket_MLA         472 SNPWSSLHKGFSA  484
OaAEP2_MLA                472 SNPWSSLHKGFSA  484
OaAEP2_pocket             472 SNPWSSLHKGFSA  484
```

Supplementary Figure 4. Alignment of *O. affinis* AEP isoforms and engineered variants. Protein sequences were aligned using ClustalW[6]. Identical residues are marked with a star and similar residues with a dot. Putative signal peptides predicted by SignalP 4.0[7] are italicized with putative propeptide cleavage sites indicated by arrows and proposed based on [8]. Italicized and bolded residues indicate putative N-terminal vacuole targeting signals as predicted by bioinformatics analysis[9]. Boxed are residues that are polymorphic between AEP isoforms PxAEP3a and PxAEP3b. The catalytic histidine and cysteine residues are highlighted in yellow. The gatekeeper residue of $OaAEP1_b$[8] is shown in green with the cysteine flanking poly-proline loop in blue. The marker of ligase activity (MLA) (this study) is shown in magenta. The residues in bold are at positions predicted to be important for AEP ligase function by the protein space modelling.

```
Butelase-1          1        MKNP-------LAILFLIATVVAVVSGIR-----DDFLRLPSQASKFFQADDNVEGTRWAVLVAGSKGYVNYR  61
Butelase-1_MLA      1        MKNP-------LAILFLIATVVAVVSGIR-----DDFLRLPSQASKFFQADDNVEGTRWAVLVAGSKGYVNYR  61
Butelase-2          1 MAVDHCFLKKKTCYYGFVLWSWMLMMSLHSKAARLNPQKEWDSVIRLPTEP---VDADTDEVGTRWAVLVAGSNGYENYR  77
                             .*         *    *. .. .  . .      *  .***..      **    *********** ** ***

Butelase-1         62 HQADVCHAYQILKKGGLKDENIIVFMYDDIAYNESNPHPGVIINHPYGSDVYKGVPKDYVGEDINPPNFYAVLLANKSAL 141
Butelase-1_MLA     62 HQADVCHAYQILKKGGLKDENIIVFMYDDIAYNESNPHPGVIINHPYGSDVYKGVPKDYVGEDINPPNFYAVLLANKSAL 141
Butelase-2         78 HQADVCHAYQLLIKGGLKEENIVVFMYDDIAWHELNPRPGVIINNPRGEDVYAGVPKDYTGEDVTAENLFAVILGDRSKV 157
                      **********.* *****.***.******** .* **.******.* * *** ****** ***..  * .**.*  .* .

Butelase-1        142 TGTGSGKVLDSGPNDHVFIYYTDHGGAGVLGMPSKPYIAASDLNDVLKKKHASGTYKSIVFYVESCESGSMFDGLLPEDH 221
Butelase-1_MLA    142 TGTGSGKVLDSGPNDHVFIYYTDHGGAGVLGMPSKPYIAASDLNDVLKKKHASGTYKSIVFYVESCESGSMFDGLLPEDH 221
Butelase-2        158 KG-GSGKVINSKPEDRIFIFYSDHGGPGVLGMPNEQILYAMDFIDVLKKKHASGGYREMVIYVEACESGSLFEGIMPKDL 236
                      * *****. * * *..**.*.**** ******    . * *  ********** *. .* ***.*****.*.*..* *

Butelase-1        222 NIYVMGASDTGESSWVTYCPLQHPSPPPEYDVCVGDLFSVAWLEDCDVHNLQTETFQQQYEVVKNKT-IVALIEDGTHVV 300
Butelase-1_MLA    222 NIYVMGASDTGESSWVTYCPLQHPSPPPEYDVCVGDLFSVAWLEDCDVHNLQTETFQQQYEVVKNKTSNFKDYAMGTHVV 300
Butelase-2        237 NVFVTTASNAQENSWGTYCPGTEPSPPPEYTTCLGDLYSVAWMEDSESHNLRRETVNQQYRSVKERTSNFKDYAMGSHVM 316
                      *..*  ** . * ** ****   *******  *.***.****.**.. ***. ** .***  ** .*        *.**.

Butelase-1        301 QYGDVGLSKQTLFVYMGTDPANDNNTFTDKNSLGTPRKAVSQRDADLIHYWEKYRRAPEGSSRKAEAKKQLREVMAHRMH 380
Butelase-1_MLA    301 QYGDVGLSKQTLFVYMGTDPANDNNTFTDKNSLGTPRKAVSQRDADLIHYWEKYRRAPEGSSRKAEAKKQLREVMAHRMH 380
Butelase-2        317 QYGDTNITAEKLYLFQGFDPATVN-LPPHNGRIEAKMEVVHQRDAELLFMWQMYQRSNHLLGKKTHILKQIAETVKHRNH 395
                      **** ... . *... * ***. *      . . .    * ****.*.  *. *.*.     .*.   **. *  . ** *

Butelase-1        381 IDNSVKHIGKLLFGIEKGHKMLNNVRPAGLPVVDDWDCFKTLIRTFETHCGSLSEYGMKHMRSFANLCNAGIRKEQMAEA 460
Butelase-1_MLA    381 IDNSVKHIGKLLFGIEKGHKMLNNVRPAGLPVVDDWDCFKTLIRTFETHCGSLSEYGMKHMRSFANLCNAGIRKEQMAEA 460
Butelase-2        396 LDGSVELIGVLLYGPGKGSPVLQSVRDPGLPLVDNWACLKSMVRVFESHCGSLTQYGMKHMRAFANICNSGVSESSMEEA 475
                      .*.**  ** **.*  **  .*. **  ***.** * * *...* **.*****..*******.***.**.*.    * **

Butelase-1        461 SAQACVSIPDNPWSSLHAGFSV* 483
Butelase-1_MLA    461 SAQACVSIPDNPWSSLHAGFSV* 483
Butelase-2        476 CMVACGGHDAGHLHPSKRGYIA* 498
                .        **     .     . *.  *
```

Supplementary Figure 5. Alignment of *C. ternatea* AEP isoforms and engineered variants. Protein sequences were aligned using ClustalW[6]. Identical residues are marked with a star and similar residues with a dot. Putative signal peptides predicted by SignalP 4.0[7] are italicized with putative propeptide cleavage sites indicated by arrows and proposed based on[8]. Boxed residues indicate putative N-terminal vacuole targeting signals as predicted by[9]. The catalytic histidine and cysteine residues are highlighted in yellow. The residue homologous to the gatekeeper residue of OaAEP1$_b$[8] is shown in green with the cysteine flanking poly-proline loop in blue. The marker of ligase activity (MLA) (this study) is shown in magenta.

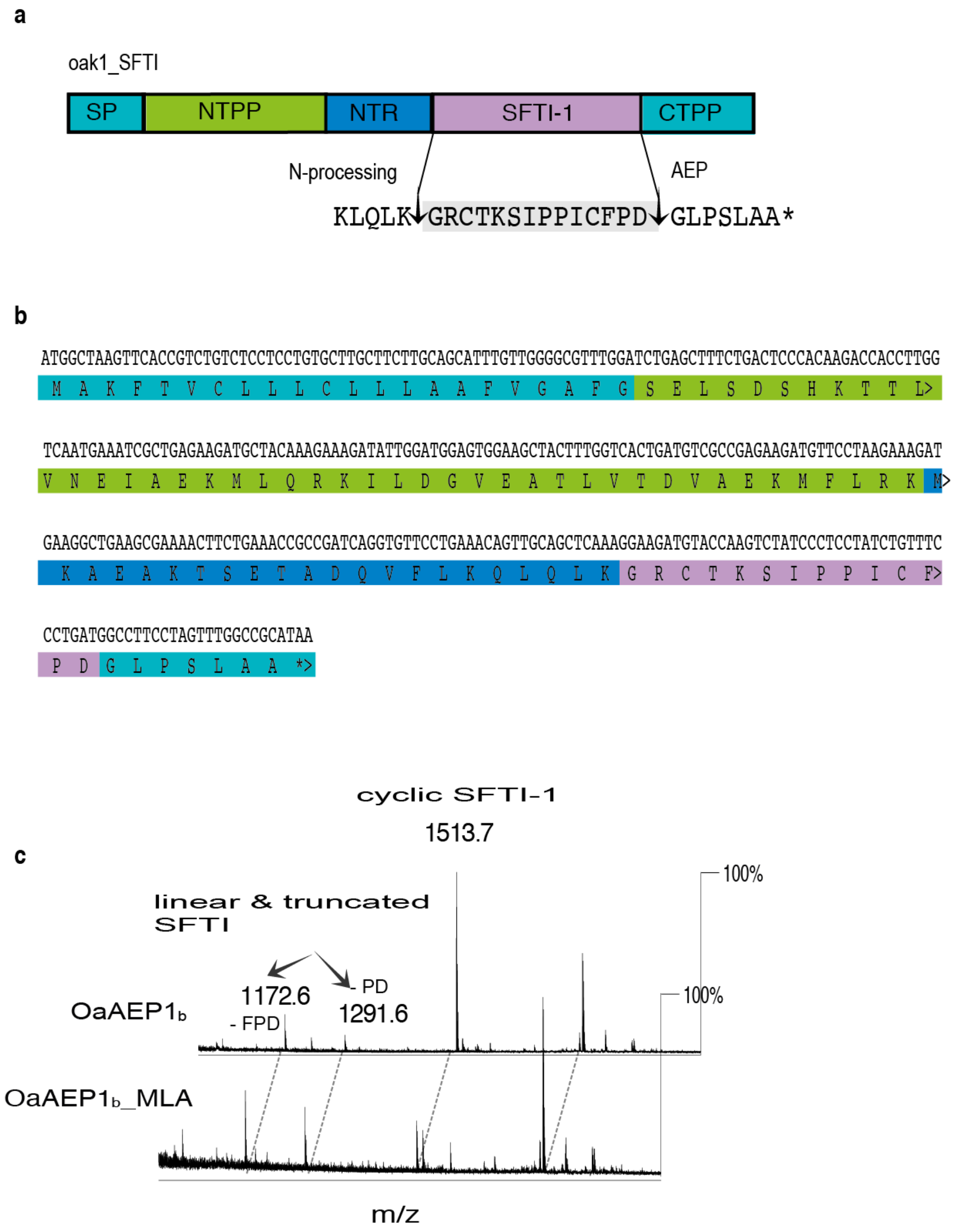

Supplementary Figure 6. AEP mediated SFTI-1 cyclisation in *N benthamiana* leaves. a, Schematic of the *Oak1* precursor gene modified to encode the SFTI peptide in replace of kB1. **b,** DNA and protein sequence of *Oak1-SFTI.* **c,** Representative MALDI-MS of peptides produced in *N. benthamiana* leaf upon co-expression of *Oak1-SFTI* with OaAEP1_b and OaAEP1_b_MLA. Cyclic SFTI was readily detected in the case of OaAEP1_b but not with OaAEP1_b_MLA. In either case no linear full length SFTI could be detected however masses for the C-terminal truncated peptides –PD (1291.6 m/z) and –FPD (1172.6 m/z) were observed.

a

```
p15_OaAEP1b       1 MHHHHHHHHLVPRGSARDGDYLHLPSEVSRFFRPQETNDDHGEDSVGTRWAVLIAGSKGYANYRHQAGVCHAYQILKRGG  80
P15_OaAEP1b_MLA   1 MHHHHHHHHLVPRGSARDGDYLHLPSEVSRFFRPQETNDDHGEDSVGTRWAVLIAGSKGYANYRHQAGVCHAYQILKRGG  80
                    ********************************************************************************

p15_OaAEP1b      81 LKDENIVVFMYDDIAYNESNPRPGVIINSPHGSDVYAGVPKDYTGEEVNAKNFLAAILGNKSAITGGSGKVVDSGPNDHI 160
P15_OaAEP1b_MLA  81 LKDENIVVFMYDDIAYNESNPRPGVIINSPHGSDVYAGVPKDYTGEEVNAKNFLAAILGNKSAITGGSGKVVDSGPNDHI 160
                    ********************************************************************************

p15_OaAEP1b     161 FIYYTDHGAAGVIGMPSKPYLYADELNDALKKKHASGTYKSLVFYLEACESGSMFEGILPEDLNIYALTSTNTTESSWCY 240
P15_OaAEP1b_MLA 161 FIYYTDHGAAGVIGMPSKPYLYADELNDALKKKHASGTYKSLVFYLEACESGSMFEGILPEDLNIYALTSTNTTESSWCY 240
                    ********************************************************************************

p15_OaAEP1b     241 YCPAQENPPPPEYNVCLGDLFSVAWLEDSDVQNSWYETLNQQYHHVDKRIS-----HASHATQYGNLKLGEEGLFVYMGS 315
P15_OaAEP1b_MLA 241 YCPAQENPPPPEYNVCLGDLFSVAWLEDSDVQNSWYETLNQQYHLVKARTSNGNSAYASHATQYGNLKLGEEGLFVYMGS 320
                    ******************************************** *  * *      ***********************

p15_OaAEP1b     316 NPANDNYTSLDGNALTPSSIVVNQRDADLLHLWEKFRKAPEGSARKEVAQTQIFKAMSHRVHIDSSIKLIGKLLFGIEKC 395
P15_OaAEP1b_MLA 321 NPANDNYTSLDGNALTPSSIVVNQRDADLLHLWEKFRKAPEGSARKEEAQTQIFKAMSHRVHIDSSIKLIGKLLFGIEKC 400
                    *********************************************** ********************************

p15_OaAEP1b     396 TEILNAVRPAGQPLVDDWACLRSLVGTFETHCGSLSEYGMRHTRTIANICNAGISEEQMAEAASQACASIP* 467
P15_OaAEP1b_MLA 401 TEILNAVRPAGQPLVDDWACLRSLVGTFETHCGSLSEYGMRHTRTIANICNAGISEEQMAEAASQACASIP  471
                    ************************************************************************
```

.

b

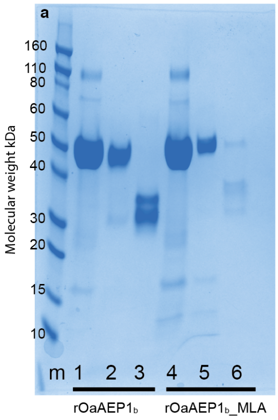

Supplementary Figure 7. Recombinant production of OaAEP1$_b$ and OaAEP1$_b$_MLA. a, The OaAEP1$_b$ and OaAEP1$_b$_MLA fusion protein sequences. For each, the putative signal peptide regions were removed and replaced with eight Histidine's allowing the capture of inactivated enzyme. **b**, Imidiazole at 250mM was used to elute rOaAEP1b and rOaAEP1b_MLA zymogens (lanes 1 and 4 respectively). AEP zymogens were self-activated at pH4.5 for 4 hours at 37°C followed by an overnight incubation at 4°C. Under these conditions activation appeared not complete (lanes 2 and 5) but was sufficient to enable purification of active enzyme by cation exchange chromatography (lanes 3 and 6).

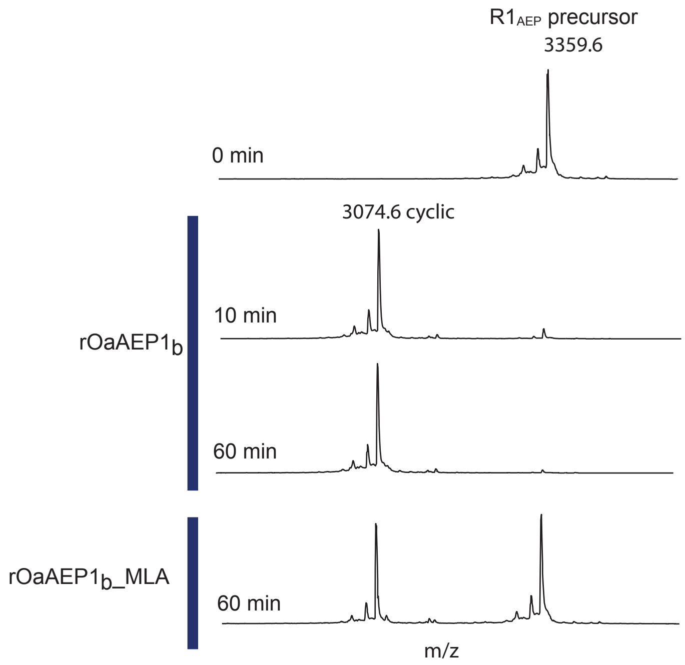

Supplementary Figure 8. *In vitro* assessment of rOaAEP1b and rOaAEP1b_MLA activity on the model peptide R1. a, Representative MALDI MS spectra of the $R1_{AEP}$ precursor peptide following incubation with recombinant $OaAEP1_b$ and $OaAEP1_b$_MLA (23.5 µg mL^{-1} total protein). For rOaAEP1b, all precursor peptide was converted to cyclic R1 (3074.6 m/z) within ten minutes, while substantial precursor peptide remained in the case of $OaAEP1_b$_MLA even after 60 minutes incubation. Observed monoiosotopic masses (Da; $[M+H]^+$) are listed.

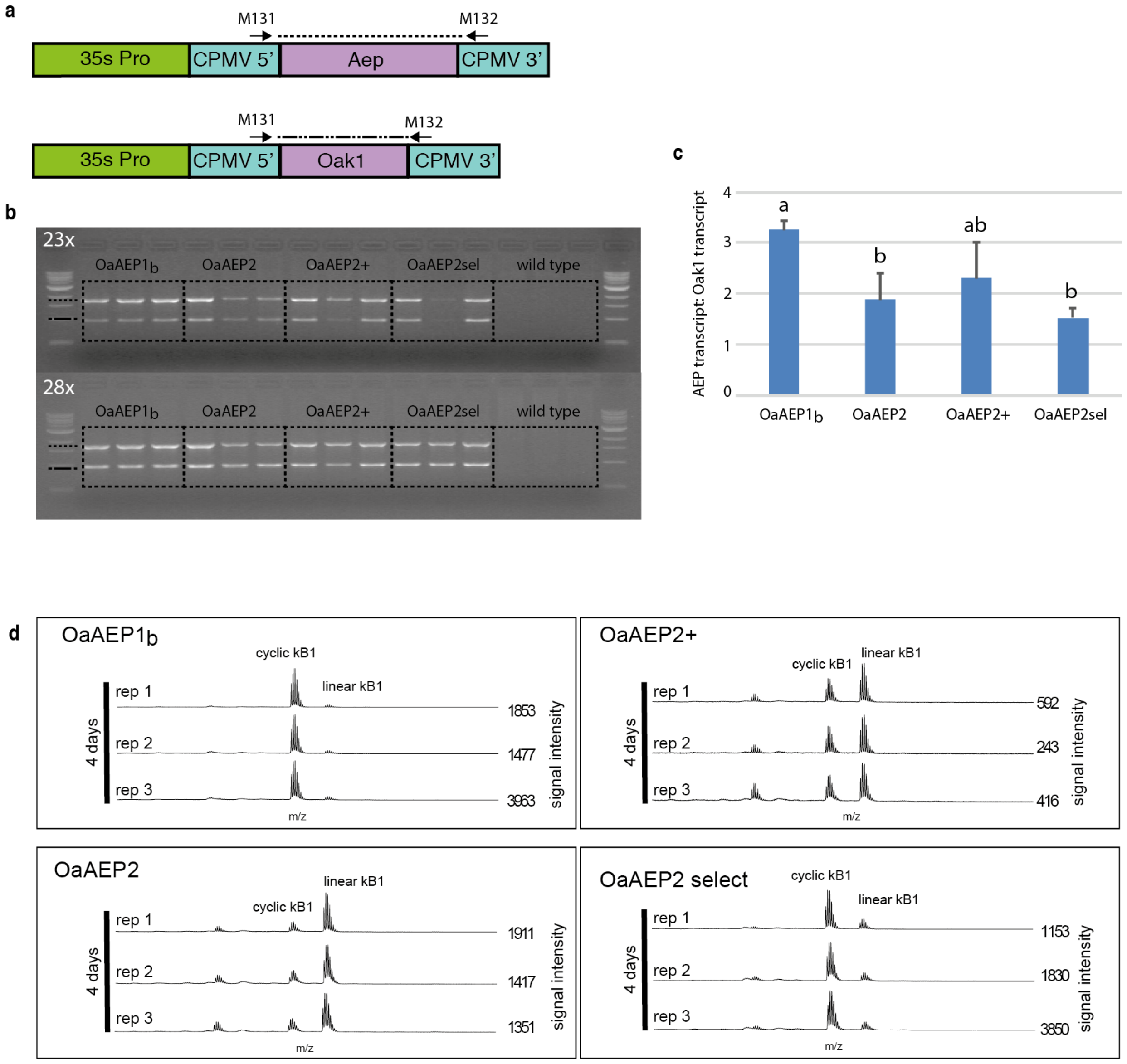

Supplementary Figure 9. AEP transgene expression levels are consistent despite significant differences in Oak1 processing. a, Plant AEP genes and *oak1* were engineered for *in planta* expression by insertion into the pEAQ-Dest1 vector [10] which contains the 35s promoter and Cowpea mosaic virus (CPMV) 5' and 3' UTR sequences. For the *N. benthamiana* leaf infiltrations, the density of Agrobacterium containing the pEAQ-Oak1 expression vector was kept constant irrespective of the co-expressed AEP. This allows an assessment of AEP transcript levels by normalizing to *Oak1* transcript. **b,** PCR analysis of *Oak1* transcript abundance (lower band) compared to AEP transcript (upper band) at PCR cycle 23 and 28 respectively. Primers M131 (5'-GACGAGGTATTGTTGCCTG-3') and M132 (5'-CCGCTCACCAAACATAG-3') were designed to bind within the 5' and 3' UTR respectively. **c,** Transcript densitometry analysis reveals a slight increase in transcript for OaAEP1$_b$, but similar levels for OaAEP2 and OaAEP2 engineered variants (OaAEP2+ and OaAEP2select) n=3. **d,** Peptide analysis at 4 days post-infiltration (n=3).

```
010G103000.1           1          MTTLVTGVFLLLLSVAGVVSAAR--YITGDVLRLPS-EASRFFRWRSDDDEVGGTRWAVLIAGSNGYWNYR  68
004G201300.1           1 MAKHDSVFIKSPSLFFLILLLFEANHIEGAGRLNQWESG-IRLPTDKDNEPEEL---DEQQVGTRWAVLVAGSSGYGNYR  76
008G236100.1           1 MAKQGYVFHNNPALLFLLLLLFEAANGGRATRLNHWESGIIRLPSEKD----------DQQLGTRWAVLVAGSSGYGNYR  70
001G023700.1           1          MTSLVVGAILLLLSLTGIVSAGR--DVTGDILRLPS-EANKFFHG-GDDDEVEGTRWAVLIAGSNGYWNYR  67
GrAEP1 009G046800.1    1          MTTLVAGVLLLLLSVTGIVTAQR--DATGDVLRLVSPEAYKFFHQ-SDDGRVGGSRWAVLIAGSRGYENYR  68
                                      .    ***        * *        .** .                 *.*****.*** ** ***

010G103000.1          69 HQADVCHAYQLLRNGGLKEENIIVFMYDDIAFNEENPRPGVIINNPHGDDVYKGVPKDYTGEDVNVHNFFAALLGNKSAI 148
004G201300.1          77 HQADVCHAYQLLRKGGLKEENIVVFMYDDIAMHELNPRPGVIINHPQGDDVYAGVPKDYTGEHVTAANLYAVLLGDKDAL 156
008G236100.1          71 HQADVCHAYQLLRKGGLKEENIIVFLYDDIAMNELNPRPGIIINHPQGDDVYAGVPKDYTGEHVTAANLYAVLLGNRSAL 150
001G023700.1          68 HQADVCHAYQLLRNGGLKEENIIVFMYDDIAYNEENPRPGIIINNPHGDDVYKGVPKDYTGENVTVNNFFAAILGNKSAL 147
GrAEP1 009G046800.1   69 HQADVCHAYQLLRKCGLKDENIVVFMYDDIAYNENNPRPGIIINSPNGSDVYHGVPKDYTGDDVTVNNFFNVILGNKAAI 148
                         *************  ***.***.**.***** .* *****.*** * * *** ********. *.  * .  .** . *.

010G103000.1         149 TGGSGKVVDSGPDDHIFIYYTDHGGPGVLGMPTFPYLYADDLIDVLKKKHASGTYKSMVFYLEACESGSIFEGLL-PQGL 227
004G201300.1         157 SGGSGKVIDSKPNDRIFMYYSDHGGPGVLGMPNMPFLYAMDFLDVLKKKHAAGTYKEMVLYVEACESGSIFEGIM-PKYL 235
008G236100.1         151 SGGSGKVVDSKTNDRIFLYYSDHGGPGVLGMPNLPFLYAMDFLDVLKKKHAAGSYREMVIYVEACESGSIFEGIM-PEDL 229
001G023700.1         148 TGGSGKVVNSGPNDHIFIYYSDHGGPGVLGMPTLPYLYADDLIDVLKKKHASGTYKSLVFYLEACESGSIFEGLL-PEGL 226
GrAEP1 009G046800.1  149 TGGSGKVVNSGPNDHIFIFYSDHGASGVLGMPDDSYIYANDLNWVLRKKHASGTYKSLVFYIEACESGSIFDGLLDPKGL 228
                         .******. *   *.**..*.***  ******   ..** *   **.****.*.*. .* *.*********.*.. *  *

010G103000.1         228 NIYATTASNAEESSWGTYCPGEYPSPPPEYETCLGDLYSVAWMEDS-DMHNLRTETLHQQYELVKKRTI--NGNSAYGSH 304
004G201300.1         236 DIYVTTASNAQESSWGTYCPGMEPPPPPEFITCLGDLYSVAWMEDS-ETHNLKRETVEQQYESVKERTSNFNADAFGGSH 314
008G236100.1         230 NIYVTTASNAQESSWGTYCPGMEPSPPPEFITCLGDLYSVAWMEDS-ETHNLKRETIEQQYKTVRERTSNFNSYTSGGSH 308
001G023700.1         227 NIYATTAANAVESSWGTYCPGEYPSPPPEYETCLGDLYSVAWMEDSSDIHNLRKETLHQQYEFVKRRTI--NGNSAYGSH 304
GrAEP1 009G046800.1  229 NIYATTASNATESSWATYCPGGQPSAPPEYDTCLGDLYSVAWIEDS-EAHDPRTETLQQQYQNVKKR--------ATTSH 299
                          ** ***.** **** *****  *  ***. ***********.***   *  . **. ***  *. *           **

010G103000.1         305 VMQYGDVGLSKDSLFAYLGTNPANDNFTFVDENSLVPPTKAVNQRDADLVHFWYKYRKAPEGSVRKTEAQKQFVEAMSHR 384
004G201300.1         315 VMEYGNTSIKPEKLYLYQGFDPATVNLP-PNELGPNTPTEAVNQRDADILFLWHMYKNSEDG-LKKTEILKQITETKRHR 392
008G236100.1         309 VMEYGSTSIKAEKLYLYQGFDPASVNFP-PNELSHDTQMEAINQRDADILFLWHMYKNSEDG-SKKEILKQISETMRHR 386
001G023700.1         305 VMQFGDIGISMDNLFTYLGTNPANDNFKFIDENSLLPPTKAVNQRDADLVHFWDKYRKAPDGSVRKVEAQKQVMEAMSHR 384
GrAEP1 009G046800.1  300 VMQYGDIVLSLDHLSVYFGENTAKYN--------LQPPTTAINQRDADLVHFWEKYRKAPEGSAKKAEAQKQLVEIMSHR 371
                         **..*   .  . *  * *   *  *              *.******..  *  *. . .*  .* *  **  *   **

010G103000.1         385 MHIDHSVKLIGKLLFGIERGLEVLNTVRPAGQPLVDDWKCLKKMVRTFETHCGSLAQYGMKHMRSLANICNAGIQTEQMA 464
004G201300.1         393 IHLDGSIDLIGTLLYGPAKGSAILKSVRETGLPLVDDWQCLKSVVRLFETHCGSLTQYGMKHMRAFANICNSGVSQALME 472
008G236100.1         387 IHLDGSIDLIGTVLYGPAKGSVILNTIREPGLPLVDDWQCLKSMVRLFETHCGSLTQYGMKHMRAFANICNSDVSQSAME 466
001G023700.1         385 MHVDNSIQLIGKLLFGVERGPEVLNTVRPTGQPLVDDWKCLKKMVRTFETHCGSLAQYGMKHMRSLANICNAGIETEKMG 464
GrAEP1 009G046800.1  372 MHIDTSVKLIGNLLFGTEIGPDVLNVVRPAGQPLVDDWKCLKEMVKTFETHCGKLAQYGMKYIRSFANICNAGIQIEHMA 451
                         .*.* *. *** .*.*   *  .*  .*  * ******.*** .*. ****** *.***** .*. *****. .    *

010G103000.1         465 EASAQACVS-VPTGRWSSLQKGFSA* 489
004G201300.1         473 EACTAACNG-HGPIQWYPSNQGYSA* 497
008G236100.1         467 EACVAACSSSHDPTQWHPSNNGYSA* 492
001G023700.1         465 EASAQACVN-IPSGHWGSVEKGFSA* 489
GrAEP1 009G046800.1  452 EASAQACVG-IHADH*           466
                         **.  **       .
```

Supplementary Figure 10. Alignment of *Gossypium raimondii* AEP sequences. Protein sequences were aligned using ClustalW[6]. Identical residues are marked with a star and similar residues with a dot. Putative signal peptides predicted by SignalP 4.0[7] are italicized with putative propeptide cleavage sites indicated by arrows and proposed based on[8]. Boxed residues indicate putative N-terminal vacuole targeting signals as predicted by[9]. The catalytic histidine and cysteine residues are bolded. The residue homologous to the gatekeeper residue of OaAEP1$_b$ [8] is shown in green with the cysteine flanking poly-proline loop in blue. The marker of ligase activity (MLA) (this study) is shown in magenta. Underlined are residues in homologous positions to those predicted by the protein space modelling as important for ligase function (Fig. 4c).

```
HeAEP1     1 MDVPNNSIFFFLHVIFLSVLLSSLGGQATRSSRFDPGILMPTEKQPE--AA-----------DDDEIGTRWAVLVAGSNG  67
HeAEP2     1        MTRLATGVFLLSLLAVAGISAGGRDIVDDVLLLPSDVSNFFHNNNKQTNNDDNNKDDDSTGTRWAVLIAGSNG  73
HeAEP3     1        MKLLVPGVLLLFLLALSGIAAGRP---DDFLRLPSEAAKSFLHN-----------DDDSVGTRWAVLIAGSKG  59
                    .      . *  **.  *  *      *  . .*..                  ***  *******.*** *

HeAEP1    68 YGNYRHQADVCHAYQLLRKGGLKEENIVVFMYDDIAKNELNPRPGVIINHPQGEDVYHGVPKDYTGQHVTAHNLYAVLLG 147
HeAEP2    74 YWNYRHQADVCHAYQLLKKGGLKDENIIVFMYDDIAHNFENPRPGIIINNPKGEDVYKGVPKDYTGEDVNAGNFYAVILG 153
HeAEP3    60 WQNYRHQADVCHAYQILKKGGLKDENIIVFMYDDIAYNESNPRPGIVINKPKGEDVYKGVPKDYTGENVNAVNFLAVLLA 139
               *************.*.*****.***.******** *  *****..** *.*****.********. *.* *  **.*

HeAEP1   148 NKTAVKGGSGKVVDSKPNDRIFLYYSDHGGPGVLGMPNMPYLYAMDFLEVLKKKHASKSYREMVIYVEACESGSIFEGIM 227
HeAEP2   154 NKTALTGGSGKVVNSGPNDHIFIYYTDHGGPGILGMPTSPYIYADKLVDVLKQKHASGTYKSLVFYLEACESGSIFEGLL 233
HeAEP3   140 NRSALTGGSGKVLDSGPNDRIFIYYTDHGAPVTIGMPSKPYLVAKDLVDTLKKKHAAGTYKSMVFYIESCESGSMFDGLL 219
             *..*. ******. * ***.**.**.*** *  .***  **. *    .. **.***. .*. .* *.*.*****.*.*..

HeAEP1   228 PEDLSIYVTTASNAQENSWGTYCPGEDPGAPP--EFTTCLGDLYSVAWMEDSETHNLKKETIKDQYKTVKARALRANTYH 305
HeAEP2   234 PEGLNIYATTASNAIESSWGTYCPGDHISPPP--EYETCLGDLYSVAWMEDSDVHNLRTETLHQQYELVKQRTAHSNGY- 310
HeAEP3   220 PEDANIYGMTATNSTEGSWVTYCPGQTDDYPEDDEYDVCFGDLWSVAWLEDCDAHNLRTETLDQQYEVVKKKI----EY- 294
             **   **  **.*. * ** *****     *    *.  * *** ****.**.. ***. **.  **  **  .     *

HeAEP1   306 EGSHVMEYGNRSIKGEKLYLYQGFDPATVNLP-PNNGLIDKPMEVVNQRDAELIFLWQMYKRSEDKSEKKTEILNQIKET 384
HeAEP2   311 -GSHVMQYGDVPLSKENLFLYMGTNPANENFTFVDDNSLSLPSKAVNQHDADLLHFWHKYHRAREGSSRKLEAQKEFVEM 389
HeAEP3   295 -AHIPAQYGNVSLAKDSLFVYMGTDPANDNKTFVEENTLRRPLKAVHSRDADLLHFWHKYHKAPEGTSRKIDAQKQLVEV 373
                 .**   .  . *..* *  **. *       . .    *    *. .**.*.   *. *... . . .* .   . *

HeAEP1   385 MRHRNHLDGSMELIGTLLFGPRKGSSILHSVREPGLPLVDDWKCLKSMVRLFETHCGSLTQYGMKHMRAFANICNYGISE 464
HeAEP2   390 MSHRMHLDHSVKFIGKLLFGMDEASEVLNAVRPAGNPLTDDWDCLRTLVRTFETHCGSLSQYGMKHMRSFANLCNAGISK 469
HeAEP3   374 LSHRTHVDNSIKLVGELLFGVGKASEVLNTIRPAGQPLVDDWDCLKTMVRTFETHCGSLSEYGMKHMRSFANMCNAGVQK 453
             . ** *.* *.   .* ****    *  .*...*  * ** *** **...** ********..*******.***.** *.

HeAEP1   465 ASMEEASSAACSGHDVGQWHPSVQGYSA 492
HeAEP2   470 EQMAEASSQACASFPSNPWSSLRKGFSA 497
HeAEP3   454 EQMAVAAGQACVTFPSNPWSSLDEGFSV 481
               *  *.  **      . *      *.*
```

Supplementary Figure 11. Alignment of *Hybanthus enneaspermus* AEP sequences. Protein sequences were aligned using ClustalW[6]. Identical residues are marked with a star and similar residues with a dot. Putative signal peptides predicted by SignalP 4.0[7] are italicized with putative propeptide cleavage sites indicated by arrows and proposed based on [8]. Boxed residues indicate putative N-terminal vacuole targeting signals as predicted by[9]. The catalytic histidine and cysteine residues are bolded. The residue homologous to the gatekeeper residue of OaAEP1$_b$[8] is shown in green with the cysteine flanking poly-proline loop in blue. The marker of ligase activity (MLA) (this study) is shown in magenta. Underlined are residues in homologous positions to those predicted by the protein space modelling as important for ligase function (Fig. 4c).

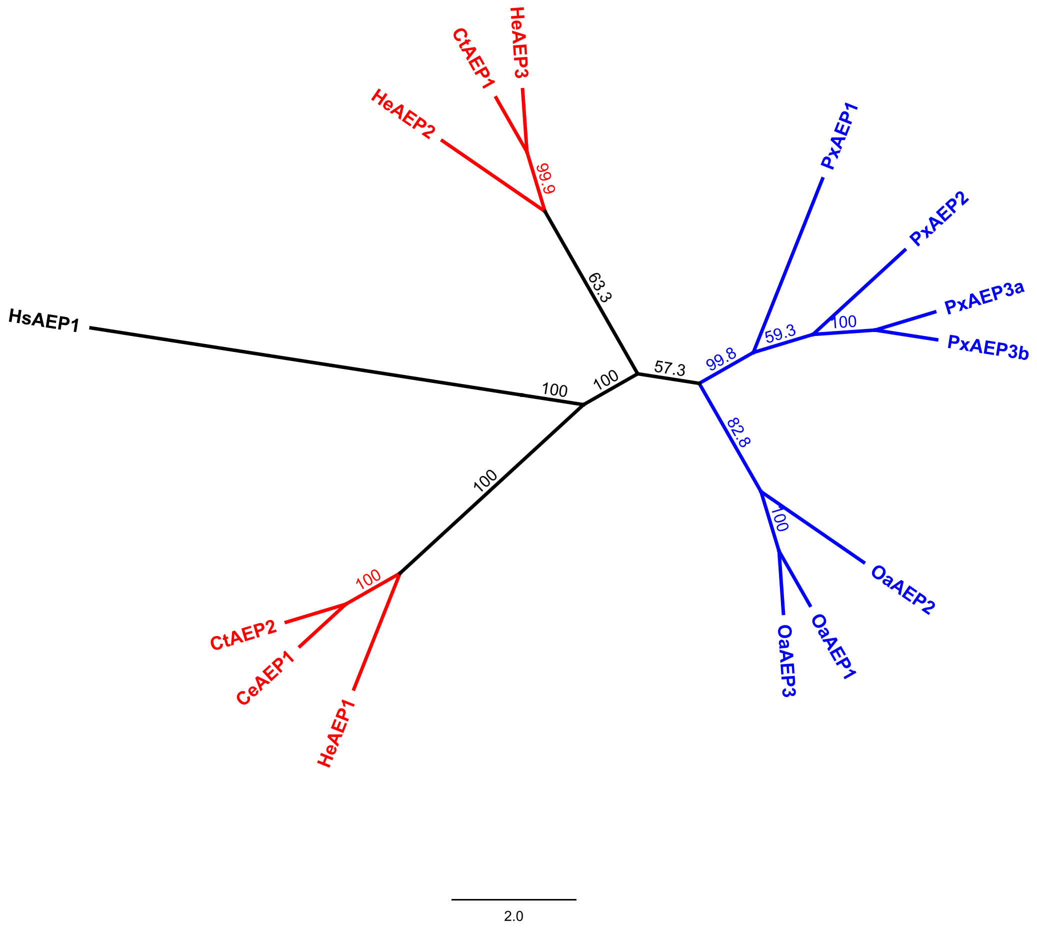

Supplementary Figure 12. Phylogentic tree of plant AEPs which differ in functional preference. AEPs are grouped into two broad groups the Asterids (red text) vs Rosids (blue text). Ligase-type and protease type AEPs are intermixed in the respective clades, especially in the Asterids (includes the Rubiaceae and Solanaceae). Ligase-type AEPs are not more closely related to one another but share greatest homology with intra-specific AEPs. The tree is rooted with human AEP (HsAEP1) and is a consensus neighbor-joining tree of 1000 bootstrapped trees.

```
        10         20         30         40         50         60         70         80         90        100
ATGGCTAAGTTCACCGTCTGTCTCCTCCTGTGCTTGCTTCTTGCAGCATTTGTTGGGGCGTTTGGATCTGAGCTTTCTGACTCCCACAAGACCACCTTGG
 M  A  K  F  T  V  C  L  L  L  C  L  L  L  A  A  F  V  G  A  F  G  S  E  L  S  D  S  H  K  T  T  L>
____________________________________TRANSLATION OF OAK KB6 [A]______________________________________>

       110        120        130        140        150        160        170        180        190        200
TCAATGAAATCGCTGAGAAGATGCTACAAAGAAAGATATTGGATGGAGTGGAAGCTACTTTGGTCACTGATGTCGCCGAGAAGATGTTCCTAAGAAAGAT
V  N  E  I  A  E  K  M  L  Q  R  K  I  L  D  G  V  E  A  T  L  V  T  D  V  A  E  K  M  F  L  R  K  M>
____________________________________TRANSLATION OF OAK KB6 [A]______________________________________>

       210        220        230        240        250        260        270        280        290        300
GAAGGCTGAAGCGAAAACTTCTGAAACCGCCGATCAGGTGTTCCTGAAACAGTTGCAGCTCAAAGGACTTCCAACATGCGGTGAGACTTGTTTCGGTGGA
  K  A  E  A  K  T  S  E  T  A  D  Q  V  F  L  K  Q  L  Q  L  K  G  L  P  T  C  G  E  T  C  F  G  G>
____________________________________TRANSLATION OF OAK KB6 [A]______________________________________>

       310        320        330        340        350        360
ACTTGCAACACTCCAGGCTGCTCTTGCTCCTCCTGGCCTATTTGCACACGCAATTAA
 T  C  N  T  P  G  C  S  C  S  S  W  P  I  C  T  R  N  *>
_________________________TRANSLATION OF OAK KB6 [A]______
```

Supplementary Figure 13. DNA and protein sequence of *Oak1-kB6trunc*.

Supplementary Table 1. RNA-seq summary statistics

RNA-seq Summary Statistics		
	Hybanthus enneaspermus	***Petunia* x *hybrida* 'Mitchell'**
**SRA accession codes**	SRP127205	SRP127205
**Pre-QC**		
Number of sequences	217735426	87112662
Sequence length	100	150
Mean PHRED score	Q36	Q37
**Post-QC**		
Number of sequences	217335174	87112662
Sequence length	90	131
Mean PHRED score	Q37	Q37
Percent of bases passing QC	89.8%	87.3%
**Assembly Statistics**		
Assembly name	HennT2.1	Px_ReTri2
Total number of bases assembled	124228394	80647009
Total number of transcripts	113823	163860
Total number of putative genes	85016	129686
Mean transcript length	1091.42	713.26

Supplementary Table 2. Functionally verified ligase and protease-type AEPs used for the protein space modelling.

		Reference
Ligases	*Oldenlandia affinis* OaAEP1 (KR259377)	*11*
	Oldenlandia affinis OaAEP1$_b$ (KR259377 with 9A-G and 1112A-T)	*12, current study*
	Oldenlandia affinis OaAEP3 (KR259378)	*12, current study*
	Oldenlandia affinis OaAEP4_.173926.	
	Clitoria ternatea Butelase-1 (KF918345)	*12, current study*
	Petunia hybrida PxAEP3b (MG720076)	*current study*
Proteases	*Oldenlandia affinis* OaAEP2 (KR259378)	*12, current study*
	Clitoria ternatea CtAEP2 (butelase-2) (KR912009)	*12, current study*
	Clitoria ternatea CtAEP6 (KY640209)	*12*
	Petunia hybrida PxAEP1 (MG720071)	*current study*
	Petunia hybrida PxAEP2 (MG720075)	*current study*
	Petunia hybrida PxAEP3a (MG720072)	*current study*
	Arabidopsis thaliana _delta (AEE76347.1)	*13*
	Arabidopsis thaliana _gamma (BAA18924.1)	*13*
	Arabidopsis thaliana _alpha (AEC07775.1)	*13*
	Arabidopsis thaliana _beta (BAA09615.1)	*13*
	*Nicotiana benthamiana*_AEP1a (BAD51740.1)	*13*
	*Nicotiana benthamiana*_AEP1b (BAD51741.1)	*13*

Supplementary Table 3. Predictive residues for AEP ligase activity. Resn$_{(MSA)}$ = residue column number in alignment, Resn$_{(ppOaAEP1)}$ = residue number in ppOaAEP1, DISORD = disorder propensity, CHRG = net static charge, RMW = molecular weight of R group, HPATH = hydropathy index.

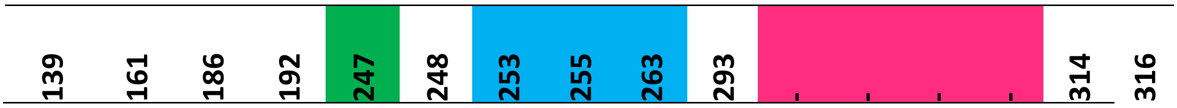

Resn$_{(MSA)}$	Resn$_{(ppOaAEP1)}$	Property	Load	PC	Ligase	Protease
**180**	**139**	CHRG	-0.18	6	K	D
**180**	**139**	CHRG	0.22	7	K	D
**219**	**161**	CHRG	-0.07	5	D	N
**274**	**186**	CHRG	0.18	7	K	G
**280**	**192**	CHRG	-0.18	5	D	N
**280**	**192**	CHRG	-0.16	7	D	N
**352**	**247**	RMW	0.07	7	C	G
**353**	**248**	RMW	0.10	7	Y	T
**359**	**253**	CHRG	0.10	5	Q	E
**359**	**253**	CHRG	0.08	7	Q	E
**361**	**255**	DISORD	-0.13	5	A	P
**379**	**263**	HPATH	0.08	5	V	T
**379**	**263**	HPATH	0.07	7	V	T
**506**	**293**	HPATH	0.21	6	H	L
**506**	**293**	RMW	-0.10	6	H	L
**506**	**293**	CHRG	-0.10	6	H	L
**506**	**293**	DISORD	-0.08	6	H	L
**506**	**293**	CHRG	-0.12	7	H	L
**506**	**293**	HPATH	0.08	7	H	L
**519**	**n/a**	NOTGAP	0.13	6	-	N
**519**	**n/a**	NOTGAP	-0.08	7	-	N
**520**	**n/a**	CHRG	0.07	5	-	G
**520**	**n/a**	NOTGAP	0.13	6	-	G
**520**	**n/a**	NOTGAP	-0.08	7	-	G
**521**	**n/a**	HPATH	0.08	5	-	N
**521**	**n/a**	NOTGAP	0.10	6	-	N
**521**	**n/a**	NOTGAP	-0.10	7	-	N
**521**	**n/a**	RMW	0.09	7	-	N
**526**	**n/a**	NOTGAP	0.13	6	-	S
**542**	**314**	CHRG	-0.19	5	E	K
**542**	**314**	HPATH	0.07	5	E	K
**542**	**314**	DISORD	-0.07	5	E	K
**544**	**316**	RMW	-0.13	5	G	K
**544**	**316**	DISORD	0.09	5	G	K
**544**	**316**	CHRG	-0.07	7	G	K

Supplementary Table 4. AEP sequences retrieved from public sequence databases that contain either a minimal MLA region or hydrophobic patch (GRAVY score > 0). Underlined are potential N-glycosylation sites and in brackets are GRAVY scores). In bold is the Gatekeeper residue homologous to Cys247 in OaAEP1$_b$.

Plant species	Accession	MLA (GRAVY)	Gate Keeper	Putative ligase
Punica Granatum	OWM76945.1	RTNASH	NSW**G**CY	no
Helianthus annuus	OTG32548.1	RIAIDKVTGFGSH (0.3)	NSW**A**TY	yes
	OTG32550.1	RIAIDKVTGFGSH (0.3)	NSW**A**TY	yes
Spinacia oleracea	KNA12580.1	RTSRMSH	SSY**A**TY	yes
Beta Vulgaris	XP_010669190.1	RTSKLSH	SSY**A**TY	yes
	KMT17971.1	RTSKLSH	SSY**A**TY	yes
Daucus carota	XP_017221562.1	RTSNDSH	DSW**A**TY	no
	KZM84774.1	RTSNDSH	DSW**A**TY	no
	KZM84775.1	RASNYSH	NSW**A**TY	no
	XP_017221563.1	RASNYSH	NSW**A**TY	no
	KZM84773.1	RTSNYSH	DSW**A**TY	no
	XP_017221560.1	RTSNYSH	DSW**A**TY	no
Eucalyptus grandis	XP_010034096.1	RTNMSH	SSY**G**YY	no
Theobroma cacao	EOY26259.1	RTAVDNLVVSSH (0.4)	NSW**G**TY	no
Gossypium raimondii	Gorai.009G046800.1	RATTSH	SSW**A**TY	yes
Malus domestica	MDP0000937205	RTNKSH	SSY**G**TY	no

Supplementary Table 5. Predictive power of the 16 most ligase-predictive residues. Known ligases are in red, and known proteases are in blue. A score of 100% would indicate that all 16 predictive residues are an identical match in the AEP sequence.

Oldenlandia affinis OaAEP1 (KR259377)	94%
Oldenlandia affinis $OaAEP1_b$ (KR259377 with 9A-G and 1112A-T)	94%
Oldenlandia affinis OaAEP3 (KR259378)	88%
Oldenlandia affinis OaAEP_4_.173926.	88%
Clitoria ternatea Butelase-1 (KF918345)	25%
Hybanthus enneaspermus HeAEP-3 (MG720074)	44%
Petunia hybrida PxAEP3b (MG720076)	38%
Oldenlandia affinis OaAEP2 (KR259378)	13%
Clitoria ternatea Butelase-2) (KR912009)	0%
Clitoria ternatea CtAEP6 (KY640209)	38%
Petunia hybrida PxAEP1 (MG720071)	0%
Petunia hybrida PxAEP2 (MG720075)	6%
Petunia hybrida PxAEP3a (MG720072)	6%
Hybanthus enneaspermus HeAEP-1 (MG720073)	6%
Hybanthus enneaspermus HeAEP-2 (MG720070)	13%
Arabidopsis thaliana _delta (AEE76347.1)	0%
Arabidopsis thaliana _gamma (BAA18924.1)	6%
Arabidopsis thaliana _alpha (AEC07775.1)	6%
Arabidopsis thaliana _beta (BAA09615.1)	0%
*Nicotiana benthamiana*_AEP1a (BAD51740.1)	6%
*Nicotiana benthamiana*_AEP1b (BAD51741.1)	0%

Supplementary Table 6. Preferred ligase residue identities for all functionally assigned AEPs with ligase prediction scores of 25% or above. Known ligases are in red, and known proteases are in blue. Predictive residues listed on the right (red if ligase predictive residue present, otherwise white). The numbering is given relative to the $OaAEP1_b$ with the Gatekeeper residue shown in green, polyproline loop residues in blue and MLA residues in magenta.

	**$Resn_{(ppOaAEP1)}$**	**139**	**161**	**186**	**192**	**247**	**248**	**253**	**255**	**263**	**293**	**n/a**	**n/a**	**n/a**	**n/a**	**314**	**316**
OaAEP1 (KR259377)	94%	1	1	1	1	1	1	1	0	1	1	1	1	1	1	1	1
OaAEP1b*	94%	1	1	1	1	1	1	1	0	1	1	1	1	1	1	1	1
OaAEP3 (KR259378)	88%	1	1	1	1	1	1	1	1	1	0	1	1	1	1	0	1
OaAEP4	88%	1	1	1	1	1	1	1	1	1	0	1	1	1	1	0	1
Butelase-1 (KF918345)	25%	0	1	1	0	0	0	1	0	1	0	0	0	0	0	0	0
HeAEP-3 (MG720074)	44%	0	1	1	0	0	0	0	0	1	0	1	1	1	1	0	0
PxAEP3b (MG720076)	38%	0	1	1	0	0	0	1	0	0	0	1	1	1	0	0	0
CtAEP6 (KY640209)	38%	0	0	0	1	0	0	0	1	0	0	1	1	1	1	0	0

*KR259377 with substitutions 9A-G and 1112A-T to produce $OaAEP1_b$

Supplementary References

1. Poth, A. G., Colgrave, M. L., Lyons, R. E., Daly, N. L. & Craik, D. J. Discovery of an unusual biosynthetic origin for circular proteins in legumes. *Proc. Natl. Acad. Sci. U. S. A.* **108**, 10127-10132 (2011).
2. Poth, A. G. *et al.* Discovery of Cyclotides in the Fabaceae Plant Family Provides New Insights into the Cyclization, Evolution, and Distribution of Circular Proteins. *ACS Chem. Biol.* **6**, 345-355 (2011).
3. Rosengren, K. J., Daly, N. L., Plan, M. R., Waine, C. & Craik, D. J. Twists, knots, and rings in proteins - Structural definition of the cyclotide framework. *J. Biol. Chem.* **278**, 8606-8616 (2003).
4. Tank, D. C. *et al.* Nested radiations and the pulse of angiosperm diversification: increased diversification rates often follow whole genome duplications. *New Phytol.* **207**, 454-467 (2015).
5. Poth, A. G. *et al.* Cyclotides associate with leaf vasculature and are the products of a novel precursor in *Petunia* (Solanaceae). *J. Biol. Chem.* **287**, 27033-27046 (2012).
6. Larkin, M. A. *et al.* Clustal W and Clustal X version 2.0. *Bioinformatics* **23**, 2947-2948 (2007).
7. Petersen, T. N., Brunak, S., von Heijne, G. & Nielsen, H. SignalP 4.0: discriminating signal peptides from transmembrane regions. *Nat. Methods* **8**, 785-786 (2011).
8. Yang, R. L. *et al.* Engineering a Catalytically Efficient Recombinant Protein Ligase. *J. Am. Chem. Soc.* **139**, 5351-5358 (2017).
9. Jackson, M. A. *et al.* A bioinformatic approach to the identification of a conserved domain in a sugarcane legumain that directs GFP to the lytic vacuole. *Functional Plant Biology* **34**, 633-644 (2007).
10. Sainsbury, F., Thuenemann, E. C. & Lomonossoff, G. P. pEAQ: versatile expression vectors for easy and quick transient expression of heterologous proteins in plants. *Plant Biotechnol. J.* **7**, 682-693 (2009).
11. Harris, K. S. *et al.* Efficient backbone cyclization of linear peptides by a recombinant asparaginyl endopeptidase. *Nat. Commun.* **6**, 10199 (2015).
12. Poon, S. *et al.* Co-expression of a cyclizing asparaginyl endopeptidase enables efficient production of cyclic peptides in planta. *J. Exp. Bot.* **69**, 633-641 (2017).
13. Gillon, A. D. *et al.* Biosynthesis of circular proteins in plants. *Plant J.* **53**, 505-515 (2008).
