# Supplementary material for: Molecular basis for the production of cyclic peptides by plant asparaginyl endopeptidases
Source: Nat Commun. 2018 Jun 20;9:2411. doi: 10.1038/s41467-018-04669-9 (PMC6010433; doi:10.1038/s41467-018-04669-9)
Supplement: Supplementary file 3 — Description of Additional Supplementary Files [file 41467_2018_4669_MOESM3_ESM.docx]

**Description of Additional Supplementary files**

File Name: Supplementary Data 1

Description: Relevant PC coordinates for protein space modelling of plant AEP sequences
